# Effects of pair migratory behavior on breeding phenology and success in a partially migratory shorebird population

**DOI:** 10.1002/ece3.9184

**Published:** 2022-08-04

**Authors:** Verónica Méndez, Jose A. Alves, Jennifer A. Gill, Böðvar Þórisson, Camilio Carneiro, Aldís E. Pálsdóttir, Sölvi R. Vignisson, Tomas G. Gunnarsson

**Affiliations:** ^1^ South Iceland Research Centre, University of Iceland Laugarvatn Iceland; ^2^ School of Biological Sciences University of East Anglia Norwich UK; ^3^ University Centre of the Westfjords Ísafjörður Iceland; ^4^ Department of Biology & CESAM—Centre for Environmental and Marine Studies University of Aveiro Aveiro Portugal; ^5^ Suðurnes Science and Learning Center Sandgerði Iceland

**Keywords:** breeding success, demography, *Haematopus ostralegus*, laying dates, oystercatcher, replacement clutches, wader

## Abstract

In migratory systems, variation in individual phenology can arise through differences in individual migratory behaviors, and this may be particularly apparent in partial migrant systems, where migrant and resident individuals are present within the same population. Links between breeding phenology and migratory behavior or success are generally investigated at the individual level. However, for breeding phenology in particular, the migratory behaviors of each member of the pair may need to be considered simultaneously, as breeding phenology will likely be constrained by timing of the pair member that arrives last, and carryover effects on breeding success may vary depending on whether pair members share the same migratory behavior or not. We used tracking of marked individuals and monitoring of breeding success from a partially migrant population of Eurasian oystercatchers (*Haematopus ostralegus*) breeding in Iceland to test whether (a) breeding phenology varied with pair migratory behavior; (b) within‐pair consistency in timing of laying differed among pair migratory behaviors; and (c) reproductive performance varied with pair migratory behavior, timing of laying, and year. We found that annual variation in timing of laying differed among pair migratory behaviors, with resident pairs being more consistent than migrant and mixed pairs, and migrant/mixed pairs breeding earlier than residents in most years but later in one (unusually cold) year. Pairs that laid early were more likely to replace their clutch after nest loss, had higher productivity and higher fledging success, independent of pair migratory behavior. Our study suggests that the links between individual migratory behavior and reproductive success can vary over time and, to a much lesser extent, with mate migratory behavior and can be mediated by differences in laying dates. Understanding these cascading effects of pair phenology on breeding success is likely to be key to predicting the impact of changing environmental conditions on migratory species.

## Graphical abstract

In avian migratory systems, variation in individual phenology can arise through differences in individual migratory behaviors, and this may be particularly apparent in partial migrant systems, where migrant and resident individuals are present within the same population. For the partially migratory Eurasian oystercatchers breeding in Iceland, we found that annual variation in timing of laying differed among pair migratory behaviors and that pairs that laid early were more likely to replace their clutch after nest loss, had higher productivity and higher fledging success, independent of pair migratory behavior. Understanding these cascading effects of pair phenology on breeding success is likely to be key to predicting the impact of changing environmental and climatic conditions on migratory species breeding in seasonal environments.

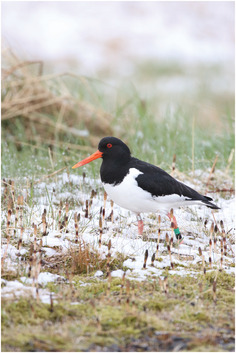

## 1 INTRODUCTION

Migration is likely to be advantageous whenever there is sufficient environmental variation to confer fitness benefits on individuals that migrate to exploit spatiotemporal variation in resource availability or quality (Boyle, 2008). Changes in environmental conditions that modify patterns of resource availability can alter the costs and benefits associated with different migratory behaviors (e.g., different migratory timings, routes, or distance). As individual migrants typically display high repeatability of migratory routes and timings (Carneiro et al., 2019b; Gill et al., 2014, 2019; Pedersen et al., 2018; Vardanis et al., 2011), quantifying the causes of any variation in fitness associated with different migratory behaviors may be key to predicting how migratory systems may respond to changing environmental conditions.

In migratory bird species, breeding success is often higher among individuals that arrive and breed early in the breeding season and lower among later breeders (Alves et al., 2019; Carneiro et al., 2021; Gunnarsson et al., 2005; Harris et al., 2006). Late arrival may constrain access to resources for breeding, particularly if these vary seasonally, and can also result in insufficient time for replacement clutches, should early nesting attempts be unsuccessful (Morrison et al., 2019). Time constraints on renesting capacity among late‐arriving individuals are likely to be particularly severe at higher latitudes where the breeding season is short (Morrison et al., 2019). Despite these apparent benefits of early arrival, timing of spring arrival can vary greatly among individuals within populations (Gill et al., 2014; Gunnarsson et al., 2004), and this variation can be related to differences in their migratory routes and distances covered (Alves et al., 2012, 2016; van Bemmelen et al., 2019). Remaining close to the breeding grounds could facilitate early arrival at the start of the breeding season (Gunnarsson & Tómasson, 2011), particularly if migration costs (in terms of energy and time) increase with migratory distance (Alves et al., 2012; Carneiro et al., 2019a; Senner et al., 2015), and/or if favorable environmental conditions at breeding areas are harder to assess from more distant locations. However, remaining close to high‐latitude breeding grounds during winter may also incur costs associated with harsher weather and limited resource availability, which may affect survival (Duriez et al., 2012) or reproductive output through carryover effects of conditions experienced during winter (Alves et al., 2013; Harrison et al., 2011). In addition, if breeding phenology of individuals with different migratory behaviors depends on environmental conditions prior to breeding and upon arrival, then fitness differences associated with those behaviors may vary over time (Harrison et al., 2013). The breeding phenology of individuals with differing migratory behaviors, and how these vary among years with differing conditions, may therefore be a key component of population responses to environmental and climate change in migratory systems (Chapman et al., 2011; Newton, 2008).

Investigating the links between individual migratory behavior, phenology, and fitness requires tracking of large numbers of individuals throughout the annual cycle and across their migratory ranges. Among the studies that have generated such data so far, the patterns reported vary considerably, suggesting that the effects of migratory behavior may be species‐ and context‐dependent. For example, individuals wintering closer to their breeding grounds typically have earlier initiation of nests and higher breeding success than individuals wintering further away in European shags *Phalacrocorax aristotelis* (Grist et al., 2017) and Eurasian spoonbills *Platalea leucorodia leucorodia* (Lok et al., 2017), but migration distance and breeding success showed no association in Great cormorants *Phalacrocorax carbo* (Bregnballe et al., 2006) and white storks *Ciconia ciconia* (Massemin‐Challet et al., 2006; Rotics et al., 2018). Conversely, in both Icelandic (*Limosa limosa islandica*) and Continental black‐tailed godwits (*L. l. limosa*), longer distance migrants can arrive and initiate their clutches earlier than shorter‐distance migrants (Alves et al., 2012; Kentie et al., 2017). These studies all investigated potential links between migratory behavior and breeding phenology or success at the individual level. However, for breeding phenology in particular, the migratory behaviors of each member of the pair may need to be considered simultaneously, as breeding phenology will likely be constrained by arrival timing of the pair member that arrives last, and carryover effects of migratory behavior on breeding success may vary depending on whether pair members share the same migratory behavior and overwinter at similar latitudes (Grist et al., 2017; Gunnarsson et al., 2004; Warkentin et al., 1990). Quantifying variation in reproductive performance in relation to pair migratory behavior, as well as individual behavior, may therefore be an important step in understanding the consequences of within‐population variation in phenology and fitness, as the relative timing of arrival of pair members may constrain the relationships between migration behavior and fitness.

We use tracking of marked individuals and intensive monitoring of breeding success from a partially migrant population of Eurasian oystercatcher (*Haematopus ostralegus*; hereafter oystercatcher) breeding across lowland Iceland, to explore the links between pair migratory behavior, breeding phenology, and breeding success. Our study was conducted between 2015 and 2018, a period that included the coldest spring recorded since 2000 (cold springs are increasingly rare at these latitudes; Alves et al., 2019; Gunnarsson et al., 2017) and three much warmer and drier years (Icelandic Meteorological Office, www.vedur.is). If spring environmental conditions are not consistent among years, then annual differences in timing of breeding are expected. This variation may differ between pairs with migrant or resident individuals, for example, if body condition upon arrival differs between individuals with distinct migratory behaviors (Harrison et al., 2013). Despite population‐level annual variation in timing of laying, pairs may consistently lay early or late within each breeding season (i.e., early‐nesting pairs may always be among the earlier‐nesters, and late‐nesting pairs may always be among the later‐nesters). However, opportunities for consistency in relative laying dates might be greater for resident pairs than pairs with one or more migrants, for which migration conditions might introduce variation in timing of arrival. Consequently, we test whether pairs with differing migratory behaviors (both resident, both migrant, or one of each) vary in (1) breeding phenology, (2) within‐pair consistency in timing of laying, and (3) reproductive performance. Finally, in order to explore consequences of variation in breeding phenology with pair migratory behavior, we also test whether (4) early‐nesting confers fitness advantages through greater nest success and/or greater capacity for replacement clutches following nest loss.

## 2 MATERIAL AND METHODS

### 2.1 Study system

Iceland supports internationally important populations of many migratory avian species, particularly waders and wildfowl (Gunnarsson, et al., 2006a). Due to its geographical location, migratory landbirds breeding in Iceland must undertake a sea crossing in excess of 800 km in order to reach wintering areas further south (Alves et al., 2012; Gunnarsson & Tómasson, 2011). The costs associated with this sea crossing may be considerable, given the absence of stop‐over sites in which to shelter if unfavorable weather conditions are encountered *en route* (Newton, 2008). A small number of Iceland‐breeding birds have populations that are partially migrant, including the oystercatcher, for which ~30% of the breeding population winters in Iceland (hereafter termed residents; but note that movements within Iceland can occur) and the remainder migrate to coastal sites throughout western Europe (hereafter migrants; Méndez et al., 2020; Þórisson et al., 2018). Iceland is at the northernmost edge of the species' distribution range, where harsher environmental conditions are likely to occur more frequently than in more southerly parts of the range. Both residents and migrants breed across Iceland, and there is no evidence of complete assortative or disassortative mating among migratory behaviors, with ~20% of pairs being resident, ~46% migrant, and ~34% mixed (comprising one resident and one migrant). Oystercatchers breed in open areas without concealing their nest, with nest‐laying dates from late April to June, including renesting following nest loss, and clutches varying between one and four eggs. They are long‐lived, with average adult survival of ~90% (Méndez et al., 2018) and establish long‐term monogamous pair bonds (van de Pol et al., 2014). Both pair members defend the breeding territory and take similar shares in incubation (Bulla et al., 2016) and parental care (van de Pol et al., 2014). Site fidelity to breeding and wintering ranges is also high (van de Pol et al., 2014). Therefore, breeding phenology and success of oystercatcher pairs can be recorded efficiently given their conspicuous nests and chicks and that most families remain in territory throughout chick development.

### 2.2 Individual tracking of Icelandic oystercatchers

Since 2013, incubating oystercatchers in the south, west, and north‐west Iceland have been captured, measured, and individually marked with colored leg rings. Adults were caught on the nest using a spring trap, and feather samples were collected for stable isotope analysis (see Méndez et al., 2020 for details).

Through a network of volunteer observers reporting sightings of marked individuals throughout the wintering range, the migratory behavior (resident or migrant) of 186 of the 537 marked individuals has been identified. For the remaining 351 individuals, migratory behavior has been determined using a discriminant function analysis of stable isotope ratios (δ^13^C and δ^15^N), after calibration using the isotopic signatures of those individuals that were observed during winter within or outside Iceland (Méndez et al., 2020). In this analysis, probabilities of being migrant or resident were calculated for each individual, and these were classified into one behavior when the probability of that behavior was at least twice the other (mean assignment probability of retained individuals was 0.94 ± 0.09 SD (range 0.67–1.00) for migrants and 0.77 ± 0.04 SD (range 0.67–0.82) for residents; Méndez et al., 2020). Note that 73 of the individuals assigned by this method in Méndez et al. (2020) have subsequently been observed in the nonbreeding season and all had been correctly assigned their migratory behavior. Only pairs in which both individuals exceeded this level of certainty (or had been observed in the nonbreeding season) were included in the analyses (see Table S1 for group‐specific sample sizes).

### 2.3 Nest monitoring and breeding data collection

Early migrants arrive in Iceland by early March, but no nesting has been recorded before mid‐April. From mid‐April each year (2015–2018), we surveyed study areas every 2–3 days to search for returning color‐marked individuals and to find and monitor pairs and nests until hatching or clutch loss. Laying date (the date when the first egg was laid) was estimated by back‐calculating from hatching dates (assuming one egg is laid per day and 28 days of incubation, starting when last egg is laid) or incubation stage, using the egg flotation method (Liebezeit et al., 2007). For each breeding attempt (including replacement clutches following clutch loss), we recorded clutch size and the outcome (successful if at least one chick hatched or failed if predated, trampled, or abandoned).

Oystercatchers remain in the vicinity of the nest after hatching their chicks and feed them throughout the growing period. Chicks were metal‐ringed just after hatching and individually marked with color rings at around 2 weeks old (when tarsus length was sufficient to fit the rings). Families were monitored every 3–4 days until all chicks were fledged or lost, allowing productivity (number of chicks fledged per pair) and fledging success (number of chicks fledged in nests where at least one egg hatched) to be recorded.

### 2.4 Data analysis

#### 2.4.1 Laying dates

First, to examine whether timing of breeding varied annually and with pair‐migratory behavior (resident, migrant or mixed), we built a linear mixed model (LMM) where laying date was modeled with year, pair migratory behavior and their interaction as fixed effects, and pair ID as a random effect. As oystercatchers can lay a replacement clutch following nest loss, we excluded data from known second breeding attempts. We also fitted the same model excluding breeding attempts from the very cold spring (2015).

We explored whether within‐pair repeatability in relative timing of laying differed among pair migratory behaviors (i.e., whether resident pairs are more or less consistent in their timing of laying than pairs with at least one migrant). We first mean‐centered laying dates in each year by subtracting the annual mean (relative laying dates) to account for annual variation in laying dates. This mean‐centering codes laying date as a deviation from the year average, which standardizes the metric among years. Following Nakagawa and Schielzeth (2010), we then performed a repeatability analysis on pairs from each migratory behavior, using a restricted dataset including only pairs with repeated measures across at least 2 years (see Table S2 for sample sizes). We used the *rptR* package (Stoffel et al., 2017) to estimate repeatability values for each pair‐level migratory behavior and their 95% CI. Repeatability varies between 0 (no consistency in laying dates) and 1 (absolute consistency in laying dates—high repeatability).

#### 2.4.2 Reproductive performance

To investigate whether reproductive performance varied with pair migratory behavior, we modeled: *clutch size* (number of eggs laid per nest) using a GLM with a Poisson error distribution and a log‐link function; *nest success* (coded as 1 for hatched nests and 0 for failed nest, which include those that were predated, trampled, or abandoned) using a GLM with binomial error distribution and logit‐link function; *renesting probability* (coded as 0 for pairs that did not renest, or 1 for pairs that renested after nest failure) using a GLM with binomial error distribution and logit‐link; *productivity* (number of chicks fledged per breeding pair, including all breeding attempts) using a GLMM with a Poisson error distribution and a log‐link function and pair ID as a random effect; and *fledging success* (number of chicks fledged in nests where at least one egg hatched) using a GLM with Poisson error distribution and a log‐link function. The random effect of pair ID was removed from the initial model as the variance component estimate was zero for clutch size, nest success, and fledging success. We first constructed all the models with migratory behavior, year, and their interaction as fixed factors and then included relative laying date of first breeding attempt to explore the contribution of variation in breeding phenology to effects of migratory behavior on reproduction. Sample sizes for each analysis are given in Tables S1. These analyses were also conducted for females and males separately, to assess whether individual migratory behavior was associated with variation in reproductive performance irrespective of mate migratory behavior.

For all analyses, we used Program R (v. 3.5.1; R Core Team, 2020) with the package *lme4* (Bates et al., 2015) and *lmerTest* (Kuznetsova et al., 2017) for mixed models. Model selection is based on Akaike information criterion adjusted for small sample size (AIC_c_) (Burnham & Anderson, 2002). Overdispersion was not detected in any of the models used.

## 3 RESULTS

### 3.1 Variation in timing of breeding

**FIGURE 1:**
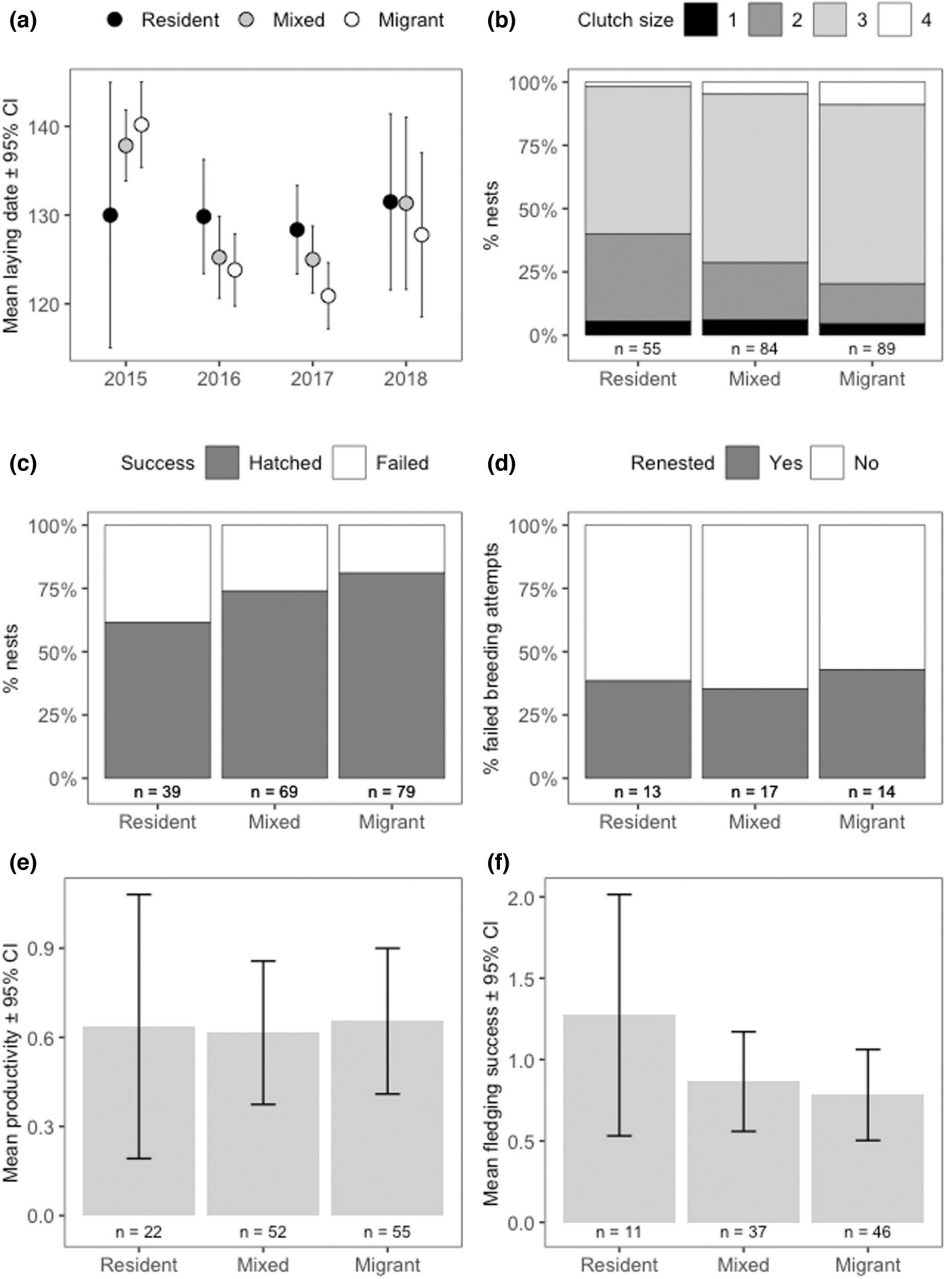
Variation in the (a) annual mean laying date (Julian date), (b) proportion of nests given the clutch size (number of eggs laid per nest), (c) proportion of nests that hatched or failed, (d) the proportion of pairs that renested after nest failure, (e) mean productivity (number of chicks fledged per pair), and (f) mean fledging success (number of chicks fledged in nests where at least one egg hatched) among pair migratory behavior. Error bars denote CIs.

**FIGURE 2:**
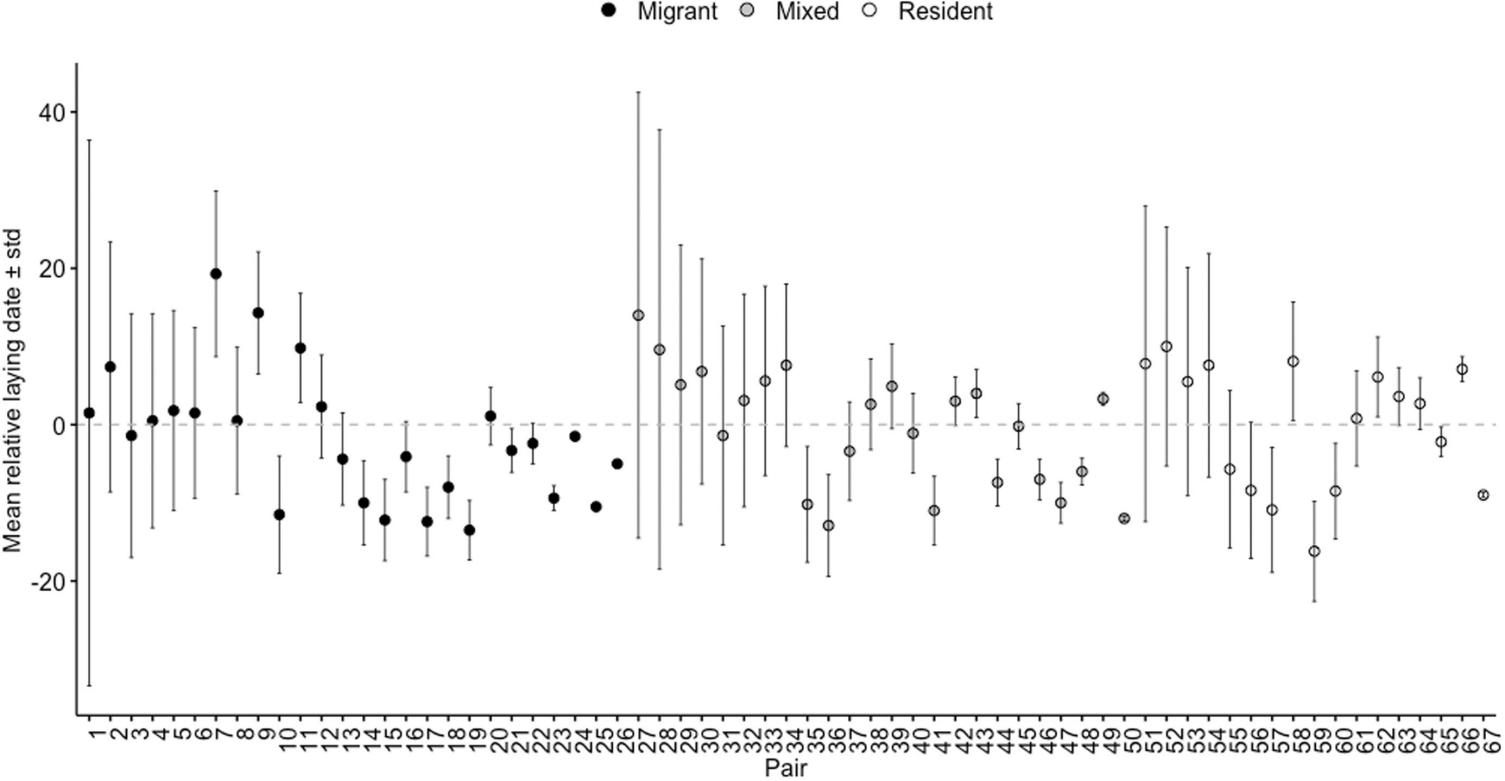
Mean (relative) lay date ± SD for oystercatcher breeding pairs, ordered by magnitude of SD (largest to smallest) within migratory behavior

The laying date for 138 pairs with known migratory behavior (comprising 56 migrant, 50 mixed, and 32 resident pairs) was estimated in one or more seasons during 2015–2018, providing a total of 228 observations. We found strong support for annual variation in timing of breeding, but not for the interaction between year and pair migratory behavior (Table S4). Laying dates were much later in 2015 (mean ± SD, 18th May ± 10.1 days) than in subsequent years (5th May ± 11.7 days, 4th May ± 10.5 days, and 10th May ± 14.99 days in 2016, 2017, and 2018, respectively; Table 1). We also found support for differences in timing of breeding among pair migratory behaviors, particularly when the cold year was excluded from the analysis (Table S4), with resident pairs laying around a week later than migrant pairs (Table 1, Figure 1a). Resident pairs showed slightly higher consistency in timing of laying (*R* = 0.35, 95% CI = [0, 0.66], *n* = 17 pairs), than migrant and mixed pairs (migrants: *R* = 0.06, 95% CI = [0, 0.37], *n* = 26 pairs; mixed: *R* = 0.05, 95% CI = [0, 0.33], *n* = 24 pairs) (Figure 2).

**TABLE 1:** Parameter estimates and profile likelihood confidence intervals of the top‐ranking model exploring variation in timing of laying of oystercatcher pairs breeding in Iceland for (a) 2015–2018 and (b) 2016–2018. Random effects estimates refer to variance. Pair ID was included as a random effect to control for repeated measures.

	Estimate	95% CI	
		Lower	Upper
(a) Entire period			
Intercept	136.99	133.12	140.85
Behavior^a^			
Mixed	1.73	−1.97	5.42
Resident	4.53	0.31	8.76
Year^b^			
2016	−12.55	−16.85	−8.25
2017	−14.39	−18.64	−10.15
2018	−8.85	−14.01	−3.69
Random effects			
*σ*_Pair ID_	22.47		
*σ*_residual_	108.51		
(b) Excluding 2015			
Intercept	123.55	120.12	126.97
Behavior^a^			
Mixed	2.71	−1.33	6.76
Resident	6.21	1.71	10.71
Year^c^			
2017	−1.62	−5.17	1.93
2018	3.63	−0.95	8.22
Random effects			
*σ*_Pair ID_	19.90		
*σ*_residual_	114.60		

^a^ Reference behavior: Migrant.

^b^ Reference year: 2015.

^c^ Reference year: 2016.

### 3.2 Variation in reproductive performance

Differences among pair migratory behaviors were found in nest success, but not in any other reproductive parameter (Table 2a, Table S5, Figure 1). When relative laying date of first breeding attempt was included in the models, we found strong support for seasonal changes in renesting probability, productivity, and fledging success, but again no evidence for differences among pair migratory behavior in renesting probability and productivity (Table 2b). Pairs with earlier nesting attempts were more likely to lay a replacement clutch after nest loss, had higher productivity and higher fledging success (Table 3, Figure S1). In addition, early‐nesters tended to have larger clutches than pairs nesting later in the season (Table 3), but this effect was weakly supported, and no differences among pair migratory behaviors were found (Table 2b). In addition to differences in nest success, we also found some support for differences among pair migratory behaviors in fledging success (Table 2b), with migrant pairs having higher nest success and lower fledging success than residents, but not mixed pairs (Figure 1c,f, Table S5). Models constructed separately for males and females showed similar patterns, but the effect of behavior on fledging success is more apparent in males (Tables S6, and S7, Figure S2), suggesting that males may play a more important role than females in protecting and provisioning chicks during the prefledging period.

**TABLE 2:** Model selection results where different parameters of reproductive performance were modeled as a function of (a) year, migration behavior, and their interaction and (b) (relative) laying date of first breeding attempt, year, and migration behavior of oystercatcher breeding pairs

		Predictors	df	logLik	AIC_c_	ΔAIC_c_	Weight
Clutch size	(a)	Null	1	−346.68	695.4	0	0.721
		Behavior	3	−346.18	698.5	3.09	0.154
		Year	4	−345.52	699.2	3.83	0.106
		Behavior + Year	6	−345.16	702.7	7.32	0.019
		Behavior × Year	12	−344.96	715.4	19.99	0
	(b)	Laying date	2	−345.18	694.4	0	0.455
		Null	1	−346.68	695.4	0.97	0.280
		Laying date + Behavior	4	−344.86	697.9	3.49	0.079
		Laying date + Year	5	−343.99	698.2	3.84	0.067
		Behavior	3	−346.18	698.5	4.06	0.06
		Year	4	−345.52	699.2	4.8	0.041
		Laying date + Behavior + Year	7	−343.79	702.1	7.69	0.010
		Behavior + Year	6	−345.16	702.7	8.29	0.007
Nest success	(a)	Year	4	−98.12	204.5	0	0.623
		Behavior + Year	6	−96.54	205.5	1.07	0.364
		Behavior × Year	12	−94.15	214.1	9.63	0.005
		Behavior	3	−103.99	214.1	9.64	0.005
		Null	1	−106.51	215	10.57	0.003
	(b)	Year	4	−98.12	204.5	0	0.439
		Behavior + Year	6	−96.54	205.5	1.07	0.257
		Laying date + Year	5	−97.89	206.1	1.64	0.194
		Laying date + Behavior + Year	7	−96.39	207.4	2.93	0.102
		Behavior	3	−103.99	214.1	9.64	0.004
		Null	1	−106.51	215	10.6	0.002
		Laying date + Behavior	4	−103.78	215.8	11.3	0.002
		Laying date	2	−106.22	216.5	12	0.001
Renesting probability	(a)	Null	1	−29.35	60.8	0	0.867
		Behavior	3	−29.26	65.1	4.32	0.1
		Year	4	−29.25	67.5	6.73	0.03
		Behavior + Year	6	−29.11	72.5	11.69	0.003
		Behavior × Year	12	−22.53	79.1	18.33	0
	(b)	Laying date	2	−24.82	53.9	0	0.817
		Laying date + Behavior	4	−24.51	58	4.12	0.104
		Laying date + Year	5	−24.08	59.7	5.82	0.044
		Null	1	−29.35	60.8	6.87	0.026
		Laying date + Behavior + Year	7	−23.73	64.6	10.7	0.004
		Behavior	3	−29.26	65.1	11.2	0.003
		Year	4	−29.25	67.5	13.6	0.001
		Behavior + Year	6	−29.11	72.5	18.6	0
Productivity	(a)	Null	2	−142.10	288.3	0	0.834
		Behavior	4	−142.08	292.5	4.2	0.102
		Year	5	−141.58	293.7	5.37	0.057
		Behavior + Year	7	−141.56	298	9.75	0.006
		Behavior × Year	12	−139.51	305.7	17.43	0
	(b)	Laying date	3	−135.99	278.2	0	0.818
		Laying date + Behavior	5	−135.77	282	3.86	0.119
		Laying date + Year	6	−135.55	283.8	5.61	0.049
		Laying date + Behavior + Year	8	−135.11	287.4	9.24	0.008
		Null	2	−142.10	288.3	10.1	0.005
		Behavior	4	−142.08	292.5	14.3	0.001
		Year	5	−141.59	293.7	15.5	0
		Behavior + Year	7	−141.56	298	19.9	0
Fledging success	(a)	Null	1	−117.41	236.9	0	0.61
		Behavior	3	−116.30	238.9	2.01	0.223
		Year	4	−115.73	239.9	3.05	0.133
		Behavior + Year	6	−114.88	242.7	5.86	0.033
		Behavior × Year	11	−112.41	250	13.19	0.001
	(b)	Laying date	2	−112.17	228.5	0	0.565
		Laying date + Behavior	4	−110.53	229.5	1.04	0.336
		Laying date + Year	5	−111.15	233	4.51	0.059
		Laying date + Behavior + Year	7	−109.65	234.6	6.14	0.026
		Null	1	−117.41	236.9	8.39	0.009
		Behavior	3	−116.30	238.9	10.4	0.003
		Year	4	−115.73	239.9	11.4	0.002
		Behavior + Year	6	−114.88	242.7	14.3	0

**TABLE 3:** Parameter estimates and profile likelihood confidence intervals of the top ranking model exploring variation in reproductive parameters of oystercatcher pairs breeding in Iceland between 2015 and 2018 (models in Table 2b). Random effect estimates refer to standard deviation. Estimates are provided on the back‐transformed scale.

Response	Predictors		Estimate	95% CI	
				Lower	Upper
Clutch size	Intercept		2.72	2.51	2.94
	Laying date		0.99	0.99	1.00
Nest success	Intercept		8.25	2.92	23.29
	Year^a^	2016	0.69	0.20	2.41
		2017	0.20	0.06	0.64
		2018	0.17	0.05	0.61
Renesting probability	Intercept		0.53	0.26	1.08
	Laying date		0.91	0.84	0.98
Productivity	Intercept		0.57	0.41	0.79
	Laying date		0.96	0.94	0.98
	Random	Pair ID	0.39		
Fledging success	Intercept		0.85	0.68	1.06
	Laying date		0.97	0.94	0.99

^a^ Reference year: 2015.

## 4 DISCUSSION

Migratory species typically have broad nonbreeding ranges within which individuals undertake different migratory routes and distances (Henningsson & Alerstam, 2005). Wintering closer to the breeding grounds may facilitate earlier spring arrival, and laying but may incur costs of harsher wintering conditions. Conversely, wintering further away may impede early spring arrival and nesting, but can promote benefits from milder wintering conditions and greater food availability (Chapman et al., 2011; Newton, 2008). Nevertheless, the costs and benefits of migrating different distances may vary among years, depending on local environmental conditions, and may lead to different consequences for pairs that have the same or contrasting migratory behaviors. We found that resident pairs nested at similar times in all studied years, but migrant and mixed pairs nested later than residents in 2015 (an unusually cold spring) and earlier in two (mixed pairs) and three (migrant pairs) of the other years. Resident birds were also slightly more consistent in relative lay dates than pairs with at least one migrant. Our results indicate that migrant pairs had higher nest success but lower fledging success than resident (but not mixed) pairs in these years. This suggests that the earlier nesting of pairs with migrants (which occurred in most years of our study) was sufficient to slightly enhance nest success but not overall productivity above that achieved by pairs with residents. The differences in hatching and fledging success with pair migratory behavior appear to be stronger for males than females, suggesting that males may play a more important role than females at the chick stage.

Differences in timing of laying can have important consequences because pairs that lay early are likely to have more time to replace their clutch following nest loss and experience higher overall reproductive performance. A simulation study demonstrated that seasonal declines in breeding success can be generated solely by early‐nesting individuals having more time to replace their clutches after nest loss, even when seasonal patterns of nest survival rates vary (Morrison et al., 2019). We found strong seasonal declines in breeding success in resident, migrant, and mixed breeding pairs, but no differences among pair migratory behavior. The reproductive performance of oystercatcher pairs is therefore enhanced by early laying, and part of this benefit of laying early is likely to be the greater time available to replace the clutch following nest loss. Replacement clutches may be less likely among late breeders because of the time and energy requirements of initiating postbreeding molt and building body condition for autumn migration and winter (Nilsson & Svenssonn, 1996). Our results suggest that reproductive performance in species breeding at high latitudes may depend more on timing of breeding rather than migratory behavior or variation in local breeding conditions, and that the time available for replacement clutches following nest loss may be a key driver of these timing effects. However, the differences in laying dates between resident, migrant, and mixed pairs of oystercatchers were not sufficient to generate differences in replacement clutch frequency in these years.

Although partial migration is widespread in nature (Chapman et al., 2011), the reproductive benefits of residency or migrancy may be context‐specific across systems (e.g., Hebblewhite & Merrill, 2011; Rolandsen et al., 2017). A recent meta‐analysis of fitness benefits of partial migration across birds, fish, mammals, and herpetofauna found that residency is more often associated with advantages in terms of survival than breeding success (Buchan et al., 2019). However, and contrary to our findings of no differences among pair migratory behaviors in reproductive success, studies of pairs of European shag (Grist et al., 2017) and merlin *Falco columbarius* (Warkentin et al., 1990) have found that resident pairs tended to raise more chicks than mixed and migrant pairs. In both cases, fitness differences were also closely linked to the timing of breeding, with pairs with a resident member laying or hatching their eggs earlier than migrants (Grist et al., 2017; Warkentin et al., 1990). In our study, we found that timing of breeding in Icelandic oystercatchers did not differ among migratory behaviors. However, resident pairs showed little variation in timing of nesting over the 4 years, while migrant and mixed pairs laid earlier than residents in some years and later in 1 year. The spring of 2015 was remarkably cold and pairs in this year nested on average 7–12 days later than in other years. However, this delayed nesting occurred in migrant and mixed pairs, and not in resident pairs, suggesting that the effect of the severe weather may have been greater on migrants than residents. Only one cold year occurred during this study, limiting our capacity to assess whether pairs with migrants consistently breed later in colder years. However, cold springs are increasingly rare in Iceland (Alves et al., 2019; Gunnarsson et al., 2017), and thus 2015 may turn out to have been one of the few remaining opportunities to reveal the dynamic nature of links between weather, migratory behavior, and breeding phenology at these latitudes (note that poor success of migrant pairs in this year precluded estimates of their productivity or fledging success in this year).

Interestingly, in 2015, the proportion of successful nests was highest, which may reflect factors such as the delayed start of laying resulting in fewer replacement clutches (which can be more likely to fail), or greater nesting synchrony across the ground‐nesting species in Iceland may have resulted in more relaxed predation pressure on oystercatchers, which typically nest before other species. When data from the cold year were excluded, our results also contrasted with those found in shags. In our population, both migrant and mixed pairs tended to lay earlier than resident pairs, which may suggest that the body condition required to reproduce depends on wintering conditions more than migration distance in Icelandic oystercatchers and perhaps also in Icelandic black‐tailed godwits, in which those wintering further south tend to arrive and initiate clutches earlier than those wintering in more northern locations (Alves et al., 2013; Gunnarsson, et al., 2006b; Kentie et al., 2017). Another possibility for the observed differences in breeding phenology is that migrants may be more time‐constrained than their resident counterparts, as they need enough time to raise their chicks, undergo postbreeding body molt, and prepare for autumn migration, hence being under higher pressure to lay as soon as conditions are favorable in spring. If annual variation in spring weather conditions influences the breeding phenology (and subsequent reproductive success) of migrants more than residents, then studies that span a range of years and conditions will be needed to identify the trade‐offs associated with different migratory behaviors, and ongoing reductions in the frequency of cold weather conditions in spring (Alves et al., 2019; Gunnarsson et al., 2017) will make these trade‐offs harder to detect.

While migrant and mixed pairs nested earlier during warm springs but later in a cold spring than resident pairs, the resident pairs tended to be more consistent in their timing of laying. If arrival time correlates with laying time (Bejarano & Jahn, 2018; Gow et al., 2019; Smith & Moore, 2005), then differences in consistency of laying dates could reflect differences in time of arrival at breeding sites between migrants and residents (Carneiro et al., 2019b). Alternatively, timing of breeding may be influenced by body condition and the time required to attain sufficient resources for egg‐laying and incubation, either or both of which may vary depending on the conditions experienced during preceding seasons (López‐Calderón et al., 2017; Rockwell et al., 2012). The earlier nesting of pairs with migrants in warmer springs (but not in the cold spring) may therefore reflect carryover effects of conditions experienced at any time prior to or at arrival on the breeding grounds. The effects of breeding phenology on reproductive success may thus be a key mechanism through which carryover effects operate in migratory systems. Advances in breeding phenology can potentially (a) increase productivity (e.g., through greater success rates of early nests and/or greater opportunities to renest following failure of early nests; Morrison et al., 2019) and (b) alter the distribution of fledging phenologies within a population, which can have important consequences for subsequent patterns of nonbreeding distribution and recruitment (e.g., Gill et al., 2019).

Understanding the impact of environmental and climatic variations, particularly extreme fluctuations during spring, is vital for improving predictions of the likely impact on the stability of species breeding at higher latitudes, where climatic changes are more pronounced. As most migratory species are distributed over very broad nonbreeding ranges and the migratory behavior of individuals can influence their subsequent breeding phenology and success. Our findings suggest that the effects of weather conditions on breeding phenology can depend on the migratory behavior of individuals and of their mates, and that the cascading effects of this phenological variation on subsequent reproductive success could be a key source of change in migratory populations in seasonal environments.

## AUTHOR CONTRIBUTIONS

**Verónica Méndez:** Conceptualization (equal); data curation (equal); formal analysis (lead); funding acquisition (lead); investigation (lead); methodology (lead); validation (equal); visualization (lead); writing – original draft (lead); writing – review and editing (equal). **Jose A. Alves:** Conceptualization (equal); funding acquisition (lead); investigation (lead); methodology (equal); supervision (equal); writing – original draft (equal); writing – review and editing (equal). **Jennifer A. Gill:** Conceptualization (equal); formal analysis (supporting); funding acquisition (lead); investigation (lead); methodology (supporting); project administration (equal); supervision (equal); writing – original draft (equal); writing – review and editing (equal). **Böðvar Þórisson:** Data curation (lead); methodology (supporting); project administration (supporting); writing – review and editing (supporting). **Camilio Carneiro:** Methodology (supporting); writing – review and editing (supporting). **Aldís E. Pálsdóttir:** Methodology (supporting); writing – review and editing (supporting). **Sölvi R. Vignisson:** Methodology (supporting); writing – review and editing (supporting). **Tomas G. Gunnarsson:** Conceptualization (equal); formal analysis (supporting); funding acquisition (lead); investigation (lead); methodology (supporting); project administration (equal); supervision (equal); writing – original draft (equal); writing – review and editing (equal).

## CONFLICT OF INTEREST

None declared.

## Supporting information


Appendix S1

## Data Availability

Data are available from the Dryad Digital Repository: https://doi.org/10.5061/dryad.8sf7m0cjn.
