# Supplementary material for: Effects of pair migratory behavior on breeding phenology and success in a partially migratory shorebird population
Source: Ecol Evol. 2022 Aug 4;12(8):e9184. doi: 10.1002/ece3.9184 (PMC9353121; doi:10.1002/ece3.9184)
Supplement: Supplementary file 1 — Appendix S1 [file ECE3-12-e9184-s001.docx]

**Supporting information**

**Table S1**. Sample sizes used in each analysis of variation in reproductive performance

|  |  | **Migrant** | | | | | **Mixed** | | | | | **Resident** | | | | |
| --- | --- | --- | --- | --- | --- | --- | --- | --- | --- | --- | --- | --- | --- | --- | --- | --- |
|  | **Pairs** | 2015 | 2016 | 2017 | 2018 | **total** | 2015 | 2016 | 2017 | 2018 | **total** | 2015 | 2016 | 2017 | 2018 | **total** |
| Clutch size | 138 | 22 | 29 | 29 | 9 | **89** | 12 | 26 | 34 | 12 | **84** | 5 | 19 | 17 | 14 | **55** |
| Nest success | 116 | 22 | 25 | 24 | 8 | **79** | 10 | 23 | 26 | 10 | **69** | 5 | 12 | 11 | 11 | **39** |
| Renesting probability | 44 |  |  |  |  | **14** |  |  |  |  | **17** |  |  |  |  | **13** |
| Productivity | 82 | 19 | 18 | 13 | 5 | **55** | 7 | 18 | 20 | 7 | **52** | 0 | 6 | 8 | 8 | **22** |
| Fledging success | 67 | 17 | 16 | 10 | 3 | **46** | 6 | 15 | 13 | 3 | **37** | 0 | 5 | 3 | 3 | **11** |

**Table S2**. List of pairs across migratory strategies for which laying dates were collected between 2015 and 2018.

| **Migrant pairs** | | | | | **Mixed pairs** | | | | | **Resident pairs** | | | | |
| --- | --- | --- | --- | --- | --- | --- | --- | --- | --- | --- | --- | --- | --- | --- |
| Pair ID | 2015 | 2016 | 2017 | 2018 | Pair ID | 2015 | 2016 | 2017 | 2018 | Pair ID | 2015 | 2016 | 2017 | 2018 |
| P13 | 1 | 1 | 1 | 1 | P118 | 1 | 1 | 1 | 1 | P154 | 1 | 1 | 1 | 1 |
| P107 | 1 | 1 | 1 |  | P41 | 1 | 1 | 1 | 1 | P28 | 1 | 1 | 1 |  |
| P55 | 1 | 1 | 1 |  | P83 | 1 | 1 | 1 | 1 | P3 | 1 | 1 | 1 |  |
| P92 | 1 | 1 | 1 |  | P115 | 1 | 1 | 1 |  | P61 | 1 |  | 1 | 1 |
| P121 | 1 | 1 |  |  | P122 | 1 | 1 | 1 |  | P53 |  | 1 | 1 | 1 |
| P127 | 1 | 1 |  |  | P148 | 1 | 1 |  |  | P15 |  | 1 | 1 |  |
| P143 | 1 | 1 |  |  | P170 | 1 | 1 |  |  | P158 |  | 1 | 1 |  |
| P173 | 1 | 1 |  |  | P114 | 1 |  | 1 |  | P189 |  | 1 | 1 |  |
| P72 | 1 | 1 |  |  | P82 | 1 |  | 1 |  | P68 |  | 1 | 1 |  |
| P175 | 1 |  | 1 | 1 | P106 |  | 1 | 1 | 1 | P138 |  | 1 |  | 1 |
| P89 | 1 |  | 1 | 1 | P9 |  | 1 | 1 | 1 | P40 |  | 1 |  | 1 |
| P187 | 1 |  | 1 |  | P136 |  | 1 | 1 |  | P79 |  | 1 |  | 1 |
| P22 | 1 |  | 1 |  | P144 |  | 1 | 1 |  | P134 |  |  | 1 | 1 |
| P24 | 1 |  | 1 |  | P171 |  | 1 | 1 |  | P149 |  |  | 1 | 1 |
| P39 | 1 |  | 1 |  | P182 |  | 1 | 1 |  | P166 |  |  | 1 | 1 |
| P108 |  | 1 | 1 |  | P26 |  | 1 | 1 |  | P5 |  |  | 1 | 1 |
| P109 |  | 1 | 1 |  | P69 |  | 1 | 1 |  | P67 |  |  | 1 | 1 |
| P132 |  | 1 | 1 |  | P11 |  |  | 1 | 1 |  |  |  |  |  |
| P159 |  | 1 | 1 |  | P119 |  |  | 1 | 1 |  |  |  |  |  |
| P23 |  | 1 | 1 |  | P145 |  |  | 1 | 1 |  |  |  |  |  |
| P74 |  | 1 | 1 |  | P213 |  |  | 1 | 1 |  |  |  |  |  |
| P180 |  | 1 |  | 1 | P30 |  |  | 1 | 1 |  |  |  |  |  |
| P140 |  |  | 1 | 1 | P47 |  |  | 1 | 1 |  |  |  |  |  |
| P181 |  |  | 1 | 1 | P7 |  |  | 1 | 1 |  |  |  |  |  |
| P76 |  |  | 1 | 1 |  |  |  |  |  |  |  |  |  |  |
| P81 |  |  | 1 | 1 |  |  |  |  |  |  |  |  |  |  |
| **Total** | **15** | **16** | **20** | **8** |  | **9** | **15** | **22** | **12** |  | **4** | **11** | **14** | **11** |

**Table S3**. Number of pairs in each study area and each year

|  |  | **Pair migratory behaviour** | | |
| --- | --- | --- | --- | --- |
| **Region** | **Year** | **Migrant** | **Mixed** | **Resident** |
| North West | 2015 | 2 | 4 | 4 |
|  | 2016 | 1 | 4 | 12 |
|  | 2017 | 0 | 3 | 8 |
|  | 2018 | 0 | 2 | 6 |
| South | 2015 | 20 | 6 | 0 |
|  | 2016 | 20 | 7 | 2 |
|  | 2017 | 19 | 7 | 0 |
|  | 2018 | 7 | 3 | 2 |
| West | 2015 | 0 | 2 | 1 |
|  | 2016 | 8 | 15 | 5 |
|  | 2017 | 10 | 24 | 9 |
|  | 2018 | 2 | 7 | 6 |

**Table S4**. Results from the model selection where egg laying date (Julian date) was modelled as a function of year and migratory behaviour of oystercatcher breeding pairs, including the interaction between migration behaviour and year for (a) the entire period (228 nests of 138 pairs) and (b) excluding 2015 (189 nests of 127 pairs). Pair ID was included as a random effect to control for repeated measures.

|  | **Predictors** | **df** | **logLik** | **AIC_c_** | **ΔAIC_c_** | **weight** |
| --- | --- | --- | --- | --- | --- | --- |
| A) **Entire period** | Pair behaviour + Year | 8 | -877.64 | 1771.9 | 0 | 0.497 |
|  | Year | 6 | -879.82 | 1772.0 | 0.09 | 0.476 |
|  | Pair behaviour × Year | 14 | -873.86 | 1777.7 | 5.76 | 0.028 |
|  | none | 3 | -900.29 | 1806.7 | 34.75 | 0 |
|  | Pair behaviour | 5 | -899.40 | 1809.1 | 37.13 | 0 |
| B) **Excluding 2015 data** | Pair behaviour + Year | 7 | -730.65 | 1475.9 | 0 | 0.514 |
|  | Pair behaviour | 5 | -733.27 | 1476.9 | 0.95 | 0.320 |
|  | Year | 5 | -734.24 | 1478.8 | 2.88 | 0.122 |
|  | none | 3 | -737.67 | 1481.5 | 5.55 | 0.032 |
|  | Pair behaviour × Year | 11 | -729.99 | 1483.5 | 7.55 | 0.012 |

**Table S5.** Parameter estimates and profile likelihood confidence intervals derived from models exploring variation in reproductive performance of oystercatcher pairs given their pair migratory behaviour. Random effects estimates refer to standard deviation. Estimates have been back-transformed.

|  |  |  | **95% CI** | |
| --- | --- | --- | --- | --- |
| **Response** | **Predictors** | **Estimate** | **Lower** | **Upper** |
| Clutch size | (Intercept) | 2.84 | 2.51 | 3.22 |
|  | Mixed | 0.95 | 0.79 | 1.14 |
|  | Resident | 0.90 | 0.73 | 1.11 |
| Nest success | (Intercept) | 4.27 | 2.43 | 7.49 |
|  | Mixed | 0.66 | 0.31 | 1.45 |
|  | Resident | 0.38 | 0.16 | 0.88 |
| Renesting probability | (Intercept) | 0.75 | 0.26 | 2.16 |
|  | Mixed | 0.73 | 0.17 | 3.11 |
|  | Resident | 0.83 | 0.18 | 3.88 |
| Productivity | (Intercept) | 0.55 | 0.34 | 0.87 |
|  | Mixed | 0.95 | 0.54 | 1.67 |
|  | Resident | 0.97 | 0.47 | 2.02 |
|  | Random: Pair ID | 0.55 |  | |
| Fledging success | (Intercept) | 0.78 | 0.56 | 1.08 |
|  | Mixed | 1.11 | 0.69 | 1.78 |
|  | Resident | 1.63 | 0.88 | 3.02 |

Reference migratory behaviour: Migrant.

**Table S6**. Model selection results where reproductive performance parameters were modelled as a function of (relative) laying date of first breeding attempt, year and migration behaviour of males and females. Number of observations for each migratory behaviour are also provided.

| **Response** | **Sex** | **Predictors** | **df** | **logLik** | **AICc** | **delta** | **weight** |
| --- | --- | --- | --- | --- | --- | --- | --- |
| **Clutch size** | **Female** | Laying date | 2 | -271.81 | 547.7 | 0 | 0.398 |
|  | 123 migrants | none | 1 | -273.28 | 548.6 | 0.88 | 0.256 |
|  | 56 residents | Behaviour + Laying date | 3 | -271.79 | 549.7 | 2.02 | 0.145 |
|  |  | Behaviour | 2 | -273.22 | 550.5 | 2.83 | 0.097 |
|  |  | Year + Laying date | 5 | -270.88 | 552.1 | 4.41 | 0.044 |
|  |  | Year | 4 | -272.23 | 552.7 | 4.99 | 0.033 |
|  |  | Behaviour + Year + Laying date | 6 | -270.87 | 554.2 | 6.53 | 0.015 |
|  |  | Behaviour + Year | 5 | -272.19 | 554.7 | 7.05 | 0.012 |
|  | **Male** | none | 1 | -260.45 | 522.9 | 0 | 0.31 |
|  | 101 migrants | Laying date | 2 | -259.44 | 523 | 0.03 | 0.305 |
|  | 70 residents | Behaviour | 2 | -260.44 | 525 | 2.03 | 0.112 |
|  |  | Behaviour + Laying date | 3 | -259.43 | 525 | 2.09 | 0.109 |
|  |  | Year | 4 | -258.94 | 526.1 | 3.19 | 0.063 |
|  |  | Year + Laying date | 5 | -257.94 | 526.2 | 3.33 | 0.059 |
|  |  | Behaviour + Year | 5 | -258.93 | 528.2 | 5.31 | 0.022 |
|  |  | Behaviour + Year + Laying date | 6 | -257.92 | 528.3 | 5.42 | 0.021 |
| **Nest success** | **Female** | Behaviour + Year | 5 | -89.57 | 189.5 | 0 | 0.652 |
|  | 120 migrants | Behaviour + Year + Laying date | 6 | -89.43 | 191.4 | 1.86 | 0.257 |
|  | 50 residents | Year | 4 | -93.07 | 194.4 | 4.88 | 0.057 |
|  |  | Year + Laying date | 5 | -93.00 | 196.4 | 6.85 | 0.021 |
|  |  | Behaviour | 2 | -97.01 | 198.1 | 8.59 | 0.009 |
|  |  | Behaviour + Laying date | 3 | -96.92 | 200 | 10.48 | 0.003 |
|  |  | none | 1 | -101.18 | 204.4 | 14.87 | 0 |
|  |  | Laying date | 2 | -101.13 | 206.3 | 16.82 | 0 |
|  | **Male** | Year | 4 | -86.90 | 182.1 | 0 | 0.381 |
|  | 100 migrants | Behaviour + Year | 5 | -86.04 | 182.5 | 0.41 | 0.31 |
|  | 62 residents | Year + Laying date | 5 | -86.75 | 183.9 | 1.84 | 0.152 |
|  |  | Behaviour + Year + Laying date | 6 | -85.69 | 183.9 | 1.87 | 0.15 |
|  |  | Behaviour | 2 | -93.58 | 191.2 | 9.18 | 0.004 |
|  |  | Behaviour + Laying date | 3 | -93.32 | 192.8 | 10.73 | 0.002 |
|  |  | none | 1 | -95.72 | 193.5 | 11.41 | 0.001 |
|  |  | Laying date | 2 | -95.66 | 195.4 | 13.35 | 0 |
| **Productivity** | **Female** | Laying date | 3 | -146.85 | 299.9 | 0 | 0.7 |
|  | 97 migrants | Behaviour + Laying date | 4 | -146.83 | 302 | 2.07 | 0.249 |
|  | 41 residents | Year + Laying date | 6 | -146.57 | 305.8 | 5.89 | 0.037 |
|  |  | Behaviour + Year + Laying date | 7 | -146.56 | 308 | 8.1 | 0.012 |
|  |  | none | 2 | -153.96 | 312 | 12.12 | 0.002 |
|  |  | Behaviour | 3 | -153.79 | 313.8 | 13.87 | 0.001 |
|  |  | Year | 5 | -153.71 | 317.9 | 17.99 | 0 |
|  |  | Behaviour + Year | 6 | -153.61 | 319.9 | 19.97 | 0 |
|  | **Male** | Laying date | 3 | -142.67 | 291.5 | 0 | 0.668 |
|  | 84 migrants | Behaviour + Laying date | 4 | -142.48 | 293.3 | 1.74 | 0.279 |
|  | 41 residents | Year + Laying date | 6 | -142.36 | 297.4 | 5.85 | 0.036 |
|  |  | Behaviour + Year + Laying date | 7 | -142.04 | 298.9 | 7.42 | 0.016 |
|  |  | none | 2 | -150.90 | 305.9 | 14.38 | 0.001 |
|  |  | Behaviour | 3 | -150.90 | 308 | 16.46 | 0 |
|  |  | Year | 5 | -150.48 | 311.4 | 19.89 | 0 |
|  |  | Behaviour + Year | 6 | -150.47 | 313.6 | 22.06 | 0 |
| **Fledging success** | **Female** | Laying date | 2 | -123.52 | 251.1 | 0 | 0.617 |
|  | 80 migrants | Behaviour + Laying date | 3 | -123.17 | 252.6 | 1.43 | 0.302 |
|  | 25 residents | Year + Laying date | 5 | -122.68 | 256 | 4.82 | 0.056 |
|  |  | Behaviour + Year + Laying date | 6 | -122.46 | 257.8 | 6.63 | 0.022 |
|  |  | none | 1 | -130.31 | 262.7 | 11.51 | 0.002 |
|  |  | Behaviour | 2 | -130.15 | 264.4 | 13.27 | 0.001 |
|  |  | Year | 4 | -128.92 | 266.2 | 15.1 | 0 |
|  |  | Behaviour + Year | 5 | -128.87 | 268.3 | 17.2 | 0 |
|  | **Male** | Behaviour + Laying date | 3 | -119.81 | 245.9 | 0 | 0.511 |
|  | 70 migrants | Laying date | 2 | -121.09 | 246.3 | 0.44 | 0.411 |
|  | 35 residents | Behaviour + Year + Laying date | 6 | -119.06 | 251 | 5.12 | 0.039 |
|  |  | Year + Laying date | 5 | -120.22 | 251 | 5.19 | 0.038 |
|  |  | none | 1 | -128.95 | 259.9 | 14.09 | 0 |
|  |  | Behaviour | 2 | -128.32 | 260.8 | 14.91 | 0 |
|  |  | Year | 4 | -127.87 | 264.1 | 18.28 | 0 |
|  |  | Behaviour + Year | 5 | -127.34 | 265.3 | 19.43 | 0 |

**Table S7**. Parameter estimates and profile likelihood confidence intervals of the female and male models exploring individual variation in reproductive performance. Estimates are derived from the first top models with migratory behaviour. Random effects estimates refer to standard deviation. Estimates have been back-transformed.

|  |  |  | **Estimate** | **2.5%** | **97.5%** | **z value** | **p** |
| --- | --- | --- | --- | --- | --- | --- | --- |
| **Nest success** | **Female** | (Intercept) | 12.66 | 3.67 | 43.62 | 4.02 | 0 |
|  |  | Behaviour Resident | 0.37 | 0.17 | 0.77 | -2.64 | 0.01 |
|  |  | Year 2016 | 0.47 | 0.12 | 1.78 | -1.06 | 0.29 |
|  |  | Year 2017 | 0.16 | 0.04 | 0.60 | -2.71 | 0.01 |
|  |  | Year 2018 | 0.14 | 0.03 | 0.61 | -2.64 | 0.01 |
|  | **Male** | (Intercept) | 10.44 | 3.12 | 34.97 | 3.80 | 0 |
|  |  | Behaviour Resident | 0.61 | 0.29 | 1.28 | -1.31 | 0.19 |
|  |  | Year 2016 | 0.57 | 0.14 | 2.31 | -0.79 | 0.43 |
|  |  | Year 2017 | 0.18 | 0.05 | 0.69 | -2.50 | 0.01 |
|  |  | Year 2018 | 0.13 | 0.03 | 0.57 | -2.72 | 0.01 |
| **Productivity** | **Female** | (Intercept) | 0.61 | 0.44 | 0.85 | -2.92 | 0 |
|  |  | Laying date | 0.96 | 0.94 | 0.98 | -3.55 | 0 |
|  |  | Behaviour Resident | 0.94 | 0.57 | 1.56 | -0.24 | 0.81 |
|  |  | Random: Pair ID | 0.36 |  |  |  |  |
|  | **Male** | (Intercept) | 0.52 | 0.36 | 0.76 | -3.45 | 0. |
|  |  | Laying date | 0.96 | 0.93 | 0.98 | -4.00 | 0 |
|  |  | Behaviour Resident | 1.16 | 0.72 | 1.87 | 0.62 | 0.54 |
|  |  | Random: Pair ID | 0.36 |  |  |  |  |
| **Fledging success** | **Female** | (Intercept) | 0.81 | 0.63 | 1.03 | -1.69 | 0.09 |
|  |  | Laying date | 0.96 | 0.94 | 0.98 | -3.52 | 0 |
|  |  | Behaviour Resident | 1.22 | 0.77 | 1.95 | 0.85 | 0.40 |
|  | **Male** | (Intercept) | 0.69 | 0.52 | 0.91 | -2.58 | 0.01 |
|  |  | Laying date | 0.96 | 0.94 | 0.98 | -3.92 | 0 |
|  |  | Behaviour Resident | 1.43 | 0.93 | 2.20 | 1.62 | 0.10 |

Reference migratory behaviour: Migrant.

Reference Year: 2015.


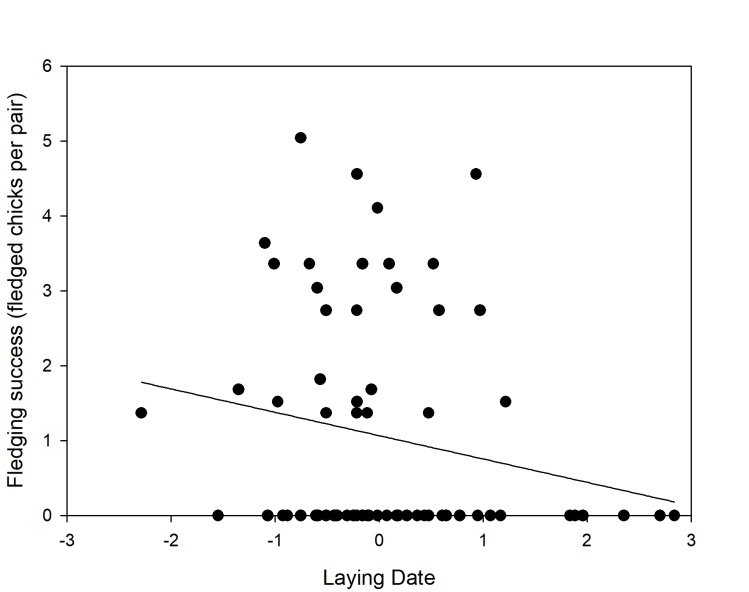


**Figure S1.** Standardised selection gradient of Oystercatchers breeding in Iceland: Laying date = -0.32 (±0.17 SE) x Fledging success + 1.07 (±0.18 SE); n = 75. Laying data was z-standardised (subtracted by mean and divide by standard deviation) and Fledging success was w-standardised (divide by mean).


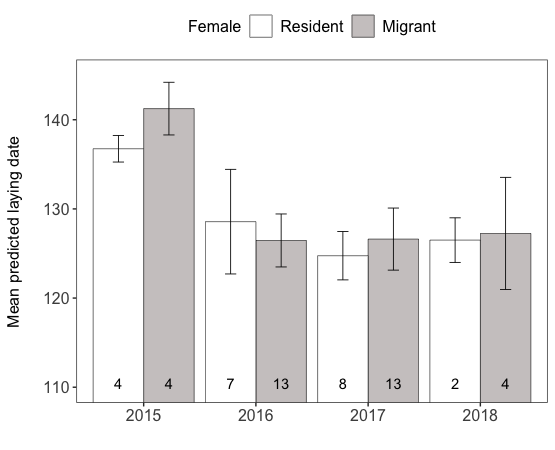


**Figure S2**. Annual variation in mean predicted Julian laying date (± SE) for migrant and resident females from mixed pairs.
